# Hippocampal–diencephalic–cingulate networks for memory and emotion: An anatomical guide

**DOI:** 10.1177/2398212817723443

**Published:** 2017-08-04

**Authors:** Emma J. Bubb, Lisa Kinnavane, John P. Aggleton

**Affiliations:** Behavioural Neuroscience Laboratory, School of Psychology, Cardiff University, Cardiff, UK

**Keywords:** Cingulate cortex, cingulum, fornix, hippocampus, mammillary bodies, Papez circuit, parahippocampal cortex, review, subiculum, thalamus

## Abstract

This review brings together current knowledge from tract tracing studies to update and reconsider those limbic connections initially highlighted by Papez for their presumed role in emotion. These connections link hippocampal and parahippocampal regions with the mammillary bodies, the anterior thalamic nuclei, and the cingulate gyrus, all structures now strongly implicated in memory functions. An additional goal of this review is to describe the routes taken by the various connections within this network. The original descriptions of these limbic connections saw their interconnecting pathways forming a serial circuit that began and finished in the hippocampal formation. It is now clear that with the exception of the mammillary bodies, these various sites are multiply interconnected with each other, including many reciprocal connections. In addition, these same connections are topographically organised, creating further subsystems. This complex pattern of connectivity helps explain the difficulty of interpreting the functional outcome of damage to any individual site within the network. For these same reasons, Papez’s initial concept of a loop beginning and ending in the hippocampal formation needs to be seen as a much more complex system of hippocampal–diencephalic–cingulate connections. The functions of these multiple interactions might be better viewed as principally providing efferent information from the posterior medial temporal lobe. Both a subcortical diencephalic route (via the fornix) and a cortical cingulate route (via retrosplenial cortex) can be distinguished. These routes provide indirect pathways for hippocampal interactions with prefrontal cortex, with the preponderance of both sets of connections arising from the more posterior hippocampal regions. These multi-stage connections complement the direct hippocampal projections to prefrontal cortex, which principally arise from the anterior hippocampus, thereby creating longitudinal functional differences along the anterior–posterior plane of the hippocampus.

## The limbic cortex, the limbic system, and the circuit of Papez

Just as the Latin word for border or edge (‘limbus’) has given us the term ‘limbo’ (that region bordering hell), so it has given us the term ‘limbic cortex’ for the cortex bordering the neocortical mantle (Broca, 1878; Da Silva et al., 1990; Pessoa and Hof, 2015). The ‘grand lobe limbique’ of Broca (1878) included the parahippocampal gyri, the underlying hippocampus, as well as the cingulate and subcallosal gyri. The subsequent notion that these structures and their interconnections play a vital role in emotion is often traced back to the work of James Papez. In ‘A proposed mechanism of emotion’, published 80 years ago, Papez (1937) tackled the daunting task of bringing together behavioural and anatomical knowledge to formulate a neuroscientific model of our emotions. With more than 3000 citations (Google Scholar), the remarkable impact of Papez’s ideas continues (Pessoa and Hof, 2015).

**Figure 1.**
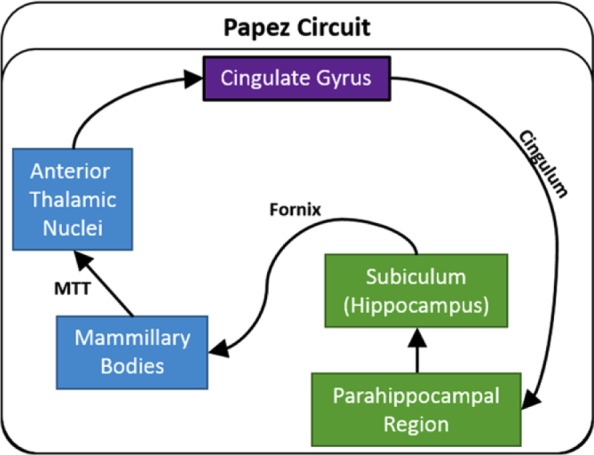
Traditional depiction of Papez circuit. The arrows show the direction of each set of connections. MTT: mammillothalamic tract.

At the heart of Papez’s model was a set of serial connections linking the hippocampus with the hypothalamus, thalamus, cingulate cortex, and back again to the hippocampus (Figure 1). The resulting circuit was thought to support and sustain emotions. While this model involved the limbic cortex of Broca, it also included key, subcortical connections within the diencephalon. Building on Papez’s ideas, Paul MacLean (1949, 1952) introduced the term ‘limbic system’. This term referred to the set of structures highlighted by Papez, but included other sites, such as the amygdala. It is MacLean’s concept of a ‘limbic system’ that has stuck, despite its many shortcomings (Isaacson, 1992; Kötter and Meyer, 1992; Roxo et al., 2011). The significance of this concept is seen in the way that limbic system connections are now regarded as vital for emotion, memory, personality, and navigation. At the same time, disruptions to these connections have been linked with numerous disorders including schizophrenia, autism, depression, obsessive-compulsive disorders, amnesia, mild cognitive impairment, and Alzheimer’s disease (Aggleton and Brown, 1999; Dalgleish, 2004; Small et al., 2011).

The purpose of this review is to re-examine those connections initially described by Papez. These core limbic connections, which are typically placed within a larger limbic system (Catani et al., 2013; Livingston and Escobar, 1971; Rolls, 2015), have particular importance for memory and spatial functions (Aggleton and Brown, 1999; Ranganath and Ritchey, 2012; Rolls, 2015; Vann et al., 2009a). Over time, this particular set of connections has been given a variety of names including Papez circuit (Teuber, 1955; Van der Horst, 1951), the Delay and Brion circuit (after Delay and Brion, 1969), the medial limbic system (Livingston and Escobar, 1971), the extended hippocampal system (Aggleton and Brown, 1999), the posterior medial temporal system (Ranganath and Ritchey, 2012), the hippocampal–diencephalic network and the parahippocampal–retrosplenial network (Catani et al., 2013), and the hippocampal limbic system (Rolls, 2015). None of these titles is ideal so we will use the term ‘hippocampal–diencephalic–cingulate network’, which reflects the key components but does not give a special weighting to just one structure.

This set of limbic connections is often thought to begin in the hippocampus (Shah et al., 2012). This notion reflects Papez’s (1937) original proposal that

“The central emotive process of cortical origin may then be conceived as being built up in the hippocampal formation and as being transferred to the mammillary body and thence through the anterior thalamic nuclei to the cortex of the gyrus cingula.” (p. 91)

Papez’s ideas were further strengthened when later tract tracing studies in animals confirmed that the direct hippocampal projections to the mammillary bodies (via the fornix) are solely efferent, as are the projections from the mammillary bodies to the anterior thalamic nuclei (via the mammillothalamic tract). These discoveries encouraged the idea of a return hippocampal loop that sequentially involved the diencephalon and cingulate cortex.

The following sections describe this hippocampal–diencephalic–cingulate network in rat, macaque (rhesus and cynomolgus monkeys), and human brains. Where possible, the routes taken by the various connections are described to help explain the effects of tract disconnections. It emerges that while the connections comprising Papez original ‘circuit’ exist as substantial pathways, there are also additional, parallel connections, as well as return projections. Together, these connections create a more complex limbic network than that often described. It should finally be added that the individual structures within this network all have numerous, additional connections beyond these limbic pathways, but these extra connections are not the focus of this review.

## The rat hippocampal–diencephalic–cingulate network

**Figure 2.**
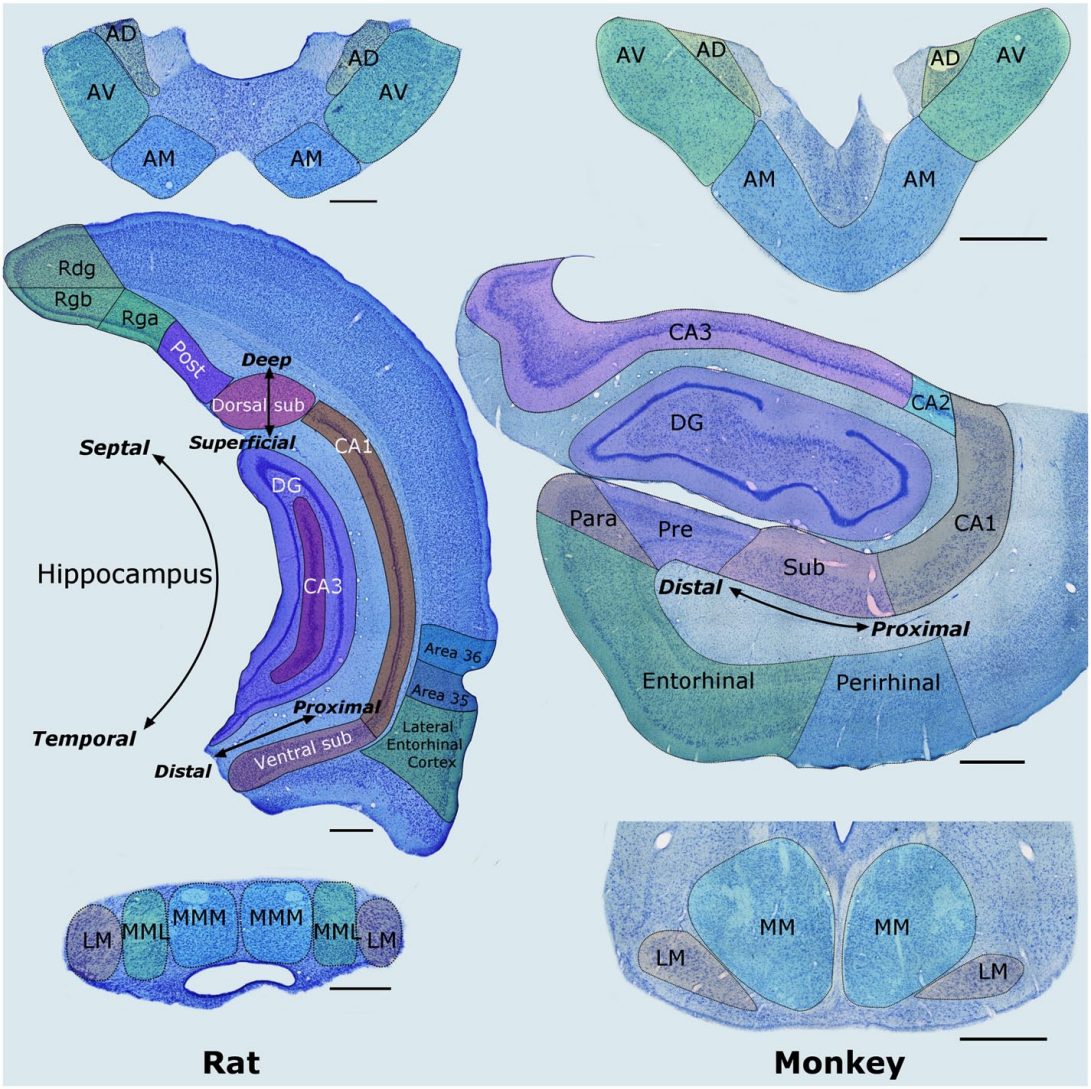
Nissl-stained coronal sections from the rat (left column) and macaque monkey (right column) showing most of the structures that comprise the hippocampal–diencephalic–cingulate network. The anterior thalamic nuclei are at the top, the hippocampus and parahippocampal region are in the middle, while the mammillary bodies are at the bottom. Left: The labels ‘Deep–Superficial’, ‘Distal–Proximal’, and ‘Septal–Temporal’ depict the three planes within the hippocampus. Scale bars = 500 µm. Right: Sections from a monkey (*Macaca fascicularis*). Scale bars = 1000 µm. AD: anterodorsal nucleus; AM: anteromedial nucleus; AV: anteroventral nucleus; CA1–3: CA fields of the hippocampus; DG: dentate gyrus; LM: lateral nucleus of the mammillary bodies; MM: medial nucleus of the mammillary bodies; MML: lateral division of the medial mammillary nucleus; MMM: medial division of the medial mammillary nucleus; Para: parasubiculum; Post: postsubiculum; Pre: presubiculum; Pro: prosubiculum; Rdg: dysgranular retrosplenial cortex (area 30); Rga: Rgb: subregions within granular retrosplenial cortex (area 29); Sub: subiculum. Note: parahippocampal areas TH and TF (and postrhinal cortex) are not depicted, neither are the monkey cingulate cortices as this would involve additional planes (see Figure 8).

Throughout this review, the term ‘hippocampus’ includes the subiculum. Adjacent to the subiculum, the postsubiculum is treated as a distinct area (Van Groen and Wyss, 1990c), even though it can be regarded as part of the presubiculum (Van Strien et al., 2009). The rat hippocampus has a ventral (‘temporal’) and dorsal (‘septal’) division (Figure 2). The rat ventral and dorsal hippocampus are, respectively, homologous with the primate anterior and posterior hippocampus (Strange et al., 2014). In addition to its long axis, the hippocampus has a medial–lateral axis, in which the ‘proximal’ subiculum borders CA1 while the ‘distal’ subiculum borders the presubiculum (Figure 2). The ‘parahippocampal region’ consists of the presubiculum, postsubiculum, parasubiculum, entorhinal cortex, perirhinal cortex (areas 35 and 36), as well as areas TH and TF (designated postrhinal cortex in the rat; Witter and Wouterlood, 2002). The connections within this network will be described in the sequence given by Papez (1937), but with new additions along the way.

### Hippocampus to mammillary bodies

These projections do not arise from the hippocampal CA fields. Rather, it is the subiculum, along with the presubiculum and postsubiculum, that provides the direct hippocampal efferents to the mammillary bodies (Allen and Hopkins, 1989; Meibach and Siegel, 1975; Swanson and Cowan, 1977; Wright et al., 2010). These subicular efferents all join the fornix before descending in the postcommissural fornix (i.e. the division of the fornix that descends behind the anterior commissure), with some fibres crossing in the columns of the fornix to reach the mammillary bodies in the opposite hemisphere.

The hippocampal projections to the mammillary bodies are topographically organised. The inputs to the medial mammillary nucleus arise from the mid-cell layer across the proximal–distal plane of the subiculum (Christiansen et al., 2016b; Naber and Witter, 1998), while the projections to the lateral mammillary nucleus arise from the postsubiculum and presubiculum (Van Groen and Wyss, 1990b, 1990c). Projections to the posterior mammillary nucleus also arise from the presubiculum (Meibach and Siegel, 1977). While the dorsal subiculum projects to dorsal parts of the medial mammillary nucleus, the ventral subiculum projects to ventral parts of the same nucleus, that is, there is a horizontal topography across the medial mammillary nucleus with respect to its hippocampal (subicular) inputs (Hopkins, 2005; Meibach and Siegel, 1975). There are, however, no direct return projections from the mammillary bodies to the hippocampus.

### Mammillary bodies to the anterior thalamic nuclei

**Figure 3.**
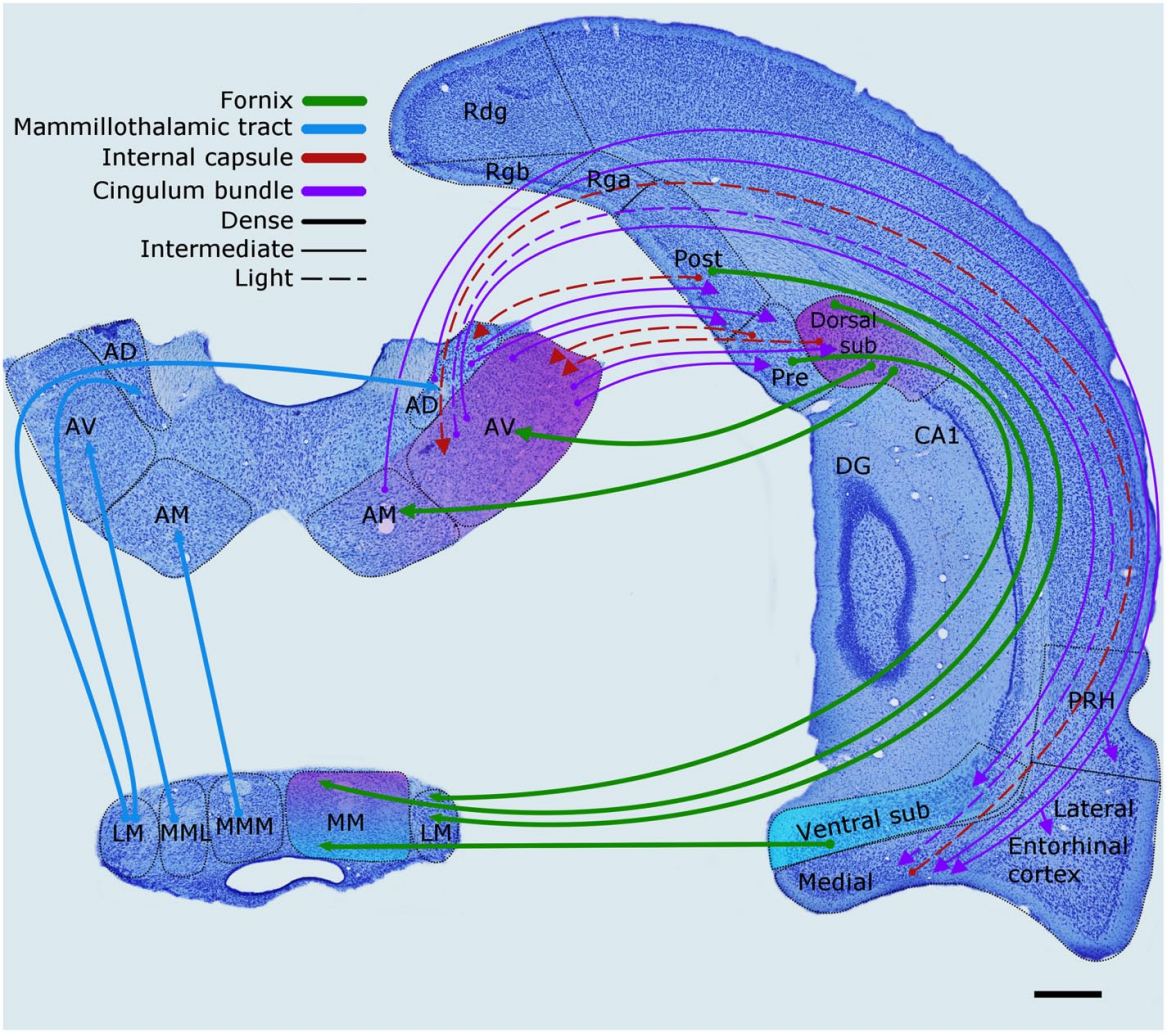
The rat brain. Depiction of the connections between the hippocampal and parahippocampal regions with the mammillary bodies and anterior thalamic nuclei, as well as the projections from the mammillary bodies to the anterior thalamic nuclei. The routes of these connections are distinguished by different colours. The origin of a connection is denoted by a circle and the termination is signified by an arrowhead, while a reciprocal connection that follows the same route has an arrowhead at both ends. The style of the lines reflects the strength of the connections (thick line = dense, thin line = intermediate, dashed line = light). The upper left panel shows the anterior thalamic nuclei, while the lower left panel depicts the mammillary bodies. AD: anterodorsal nucleus; AM: anteromedial nucleus; AV: anteroventral nucleus; CA1–3: CA fields of the hippocampus; DG: dentate gyrus; LM: lateral nucleus of the mammillary bodies; MM: medial nucleus of the mammillary bodies; MML: lateral division of the medial mammillary nucleus; MMM: medial division of the medial mammillary nucleus; Post: postsubiculum; Pre: presubiculum; PRH: perirhinal cortex; Rdg: dysgranular retrosplenial cortex (area 30); Rga: Rgb: subregions within granular retrosplenial cortex (area 29); Sub: subiculum. Scale bar = 500 µm.

The next step in this core limbic subsystem consists of the unidirectional projections from the mammillary bodies to the anterior thalamic nuclei, via the mammillothalamic tract (Figure 3). There is little evidence for interneurons in the rat mammillary bodies (Allen and Hopkins, 1988; Seki and Zyo, 1984) and it is likely that almost every mammillary body neuron contributes to this thalamic projection (Powell et al., 1957). Few, if any, mammillothalamic projections reach the laterodorsal thalamic nucleus. Thus, even though this thalamic nucleus shares many connections with the anterior thalamic nuclei, it has a separate status within this network.

The mammillary body projections consist of ipsilateral efferents from the medial mammillary nucleus to the anteroventral and anteromedial thalamic nuclei, contrasting with bilateral efferents from the lateral mammillary nucleus to the anterodorsal thalamic nucleus (Figure 3). The most midline portion of the mammillary bodies (pars medianus) projects to the most midline part of the anterior thalamic nuclei (the interoanteromedial nucleus). In general, more medial parts of the medial mammillary nucleus terminate in the anteromedial thalamic nucleus, while more lateral parts of the medial mammillary nucleus terminate in the anteroventral thalamic nucleus (Shibata, 1992). In addition, the posterior mammillary nucleus projects to a dorsal medial part of the anteroventral thalamic nucleus (Shibata, 1992). Consequently, the anterior thalamic projections from the mammillary bodies are organised in a plane largely orthogonal to the pattern of horizontal mammillary terminations from the subiculum (Hopkins, 2005). One result is that both ventral and dorsal subicular inputs to the mammillary bodies may indirectly influence the same anterior thalamic area.

Contrary to initial depictions of this limbic circuitry (Figure 1), there are dense, direct projections to the anterior thalamic nuclei from the subiculum, presubiculum, postsubiculum, and parasubiculum (Meibach and Siegel, 1977; Swanson and Cowan, 1977; Van Groen and Wyss, 1990b, 1990c). The subiculum efferents to the anteromedial nucleus rely on the fornix (Figure 3), as do the large majority of subiculum inputs to the anteroventral nucleus (Dillingham et al., 2015a). Some projections from the distal subiculum and presubiculum, however, take a parallel (nonfornical) route via the internal capsule before terminating in the dorsolateral part of the anteroventral nucleus (Dillingham et al., 2015a). Likewise, many of the hippocampal efferents to the anterodorsal nucleus, which predominantly arise from the postsubiculum and parasubiculum, project via the internal capsule (Dillingham et al., 2015a; Van Groen and Wyss, 1990b, 1990c).

The hippocampal cells giving rise to the anterior thalamic nuclei and mammillary body projections are largely segregated by their respective depths within the subiculum (Christiansen et al., 2016b; Wright et al., 2010). In addition, the proximal subiculum preferentially projects to the anteromedial nucleus, while the distal subiculum and the adjacent presubiculum preferentially project to the anteroventral thalamic nucleus (Christiansen et al., 2016b; Naber and Witter, 1998; Van Groen and Wyss, 1990b; Wright et al., 2013). These direct anterior thalamic inputs predominantly arise from the dorsal hippocampus. In contrast, the mammillary body inputs arise from both the dorsal and ventral hippocampus (Christiansen et al., 2016b).

### Anterior thalamic nuclei to cingulate cortex

**Figure 4.**
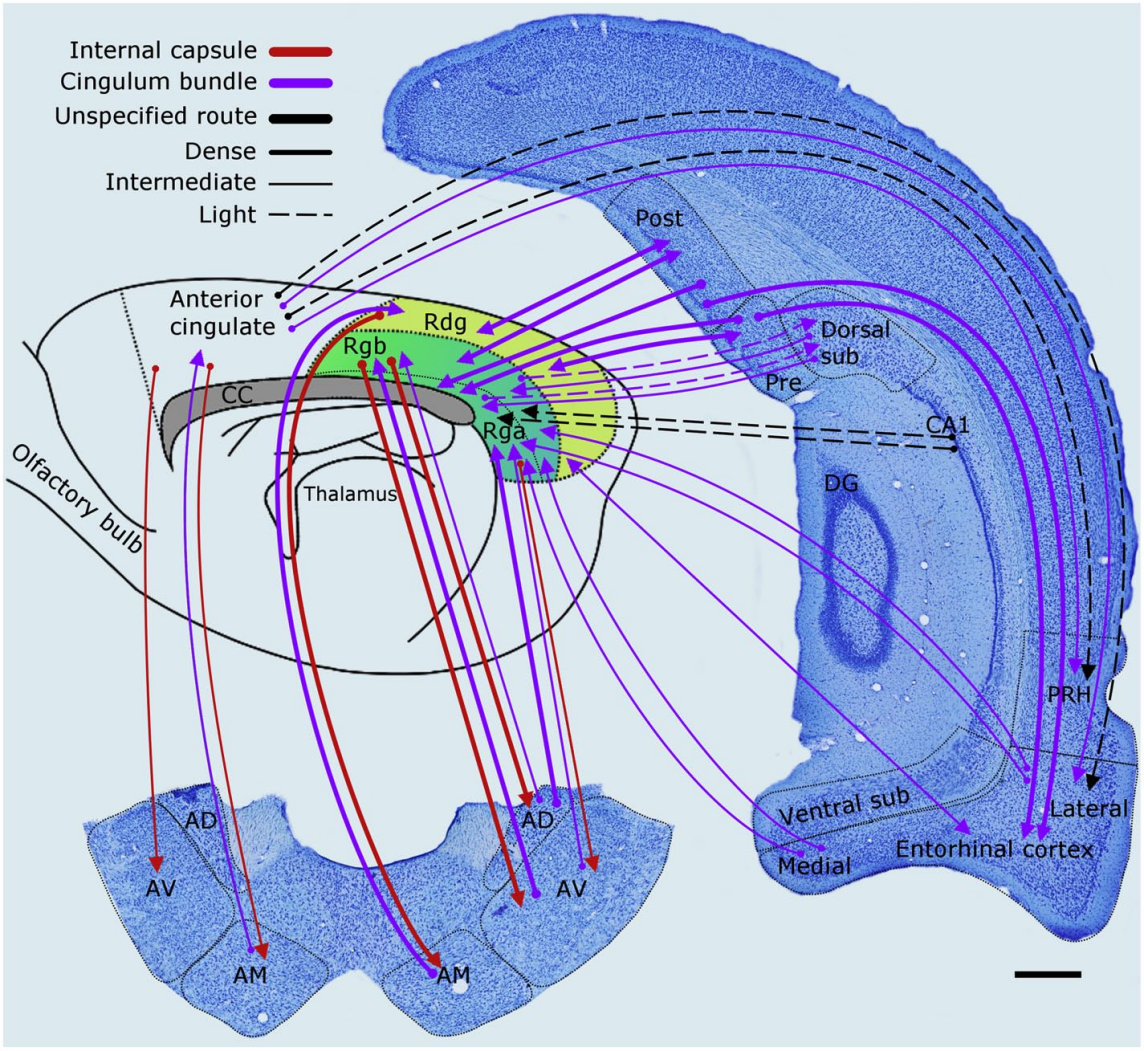
The rat brain. Depiction of the connections between the anterior thalamic nuclei (lower left), cingulate cortices, hippocampus, and parahippocampal areas. The routes of these projections are distinguished by different colours. The origin of a connection is denoted by a circle and the termination is signified by an arrowhead while a reciprocal connection that follows the same route has an arrowhead at both ends. The style of the lines reflects the strength of the connections (thick line = dense, thin line = intermediate, dashed line = light). AD: anterodorsal nucleus; AM: anteromedial nucleus; AV: anteroventral nucleus; CA1–3: CA fields of the hippocampus; CC: corpus callosum; DG: dentate gyrus; Post: postsubiculum; Pre: presubiculum; PRH: perirhinal cortex; Rdg: dysgranular retrosplenial cortex (area 30); Rga: Rgb: subregions within granular retrosplenial cortex (area 29); Sub: subiculum. Scale bar = 500 µm.

The remaining connections, which Papez regarded as unidirectional (Figure 1), are now all known to be reciprocal (Figure 4). These bidirectional connections are found between the anterior thalamic nuclei and the cingulate cortices, between the cingulate cortices and the parahippocampal region, and between the parahippocampal region and the hippocampus. In addition, there are bidirectional connections between the anterior thalamic nuclei and both the parahippocampal region and the hippocampus (Shibata, 1993). This pattern of reciprocity adds greater complexity to Papez’s initial concept of a serial circuit linking these limbic sites (Figure 4).

The first reciprocal connections to be described are between the anterior thalamic nuclei and the cingulate cortex. The rat cingulate cortex contains two major divisions, the anterior cingulate cortex (principally composed of area 24) and the posterior cingulate or, more accurately, the retrosplenial cortex (areas 29 and 30). (The term retrosplenial is more accurate as the rat brain lacks posterior cingulate areas 23 and 31, which are present in primate brains.) While multiple designations exist for the subregions within retrosplenial cortex (see Jones and Witter, 2007), we have divided granular area 29 into subregions Rga and Rgb, while the dysgranular area 30 is designated Rdg (see Van Groen and Wyss, 1990a, 1992, 2003). Within the cingulate cortex, the retrosplenial cortex has the more extensive interconnections with the anterior thalamic nuclei (Shibata, 1993), as well as appreciably denser connections with hippocampal and parahippocampal regions.

Only restricted parts of the anteromedial nucleus project to the anterior cingulate cortex (Shibata, 1993), with return projections from the same cortical area terminating in the anteromedial and anteroventral thalamic nuclei (Shibata and Naito, 2005; Wright et al., 2013). In addition, reciprocal connections with prelimbic cortex are essentially restricted to the anteromedial nucleus (Mathiasen et al., 2017; Shibata and Naito, 2005). In contrast, almost all parts of the anterior thalamic nuclei appear to project to the retrosplenial cortex (areas 29, 30), with topographical associations between a particular thalamic nucleus (and subregion) and a particular retrosplenial region (Shibata, 1993, 1998; Shibata and Kato, 1993; Van Groen and Wyss, 1990a, 1992, 2003). Both Rga and Rgb are reciprocally connected with the anteroventral nucleus while the dysgranular cortex (Rdg) has reciprocal connections with the anteromedial nucleus. Meanwhile, the anterodorsal thalamic nucleus projects to Rga and Rgb, receiving light return inputs from Rgb (Van Groen and Wyss, 1990a, 2003). The projections from the anteroventral nucleus principally terminate in layer I of Rgb while those from the anterodorsal nucleus terminate in deep II/III, as well as layer I of Rgb (Shibata, 1993; Van Groen and Wyss, 2003). The projections from the anteromedial nucleus principally terminate in layers I and V of Rdg (Shibata, 1993; Van Groen and Wyss, 1992).

The direct outputs from the anterior thalamic nuclei to the cingulate cortex are almost entirely ipsilateral, with just a small population of anteroventral cells appearing to cross to the contralateral retrosplenial cortex (Mathiasen et al., 2017). The route taken by the anterior thalamic projections often involves the cingulum bundle (Domesick, 1970), with many anterior thalamic fibres passing rostrally and then dorsally (through the striatum) before joining the bundle. Other anterior thalamic fibres emerge laterally from the anterior thalamic nuclei to reach the internal capsule, then turn dorsally to cross through the corpus callosum, and so join the cingulum more directly. Meanwhile, the efferents from the retrosplenial cortex to the anterior thalamic nuclei, which arise from layer VI (Mathiasen et al., 2017; Sripanidkulchai and Wyss 1987), reach both the ipsilateral and contralateral anterior thalamic nuclei (Mathiasen et al., 2017). The more direct route is favoured by these retrosplenial projections, that is, around the lateral ventricle, briefly joining the internal capsule, before cutting across the dorsal thalamus to reach the anterior thalamic nuclei (Shibata, 1998; Van Groen and Wyss, 1992). A small number of fibres from Rga and Rgb may reach the anterior thalamic nuclei via the fornix (Shibata, 1998).

### Cingulate cortex to the parahippocampal region and hippocampus

The retrosplenial cortex has many projections to the parahippocampal region (Figure 4), thereby completing the notional circuit (Jones and Witter, 2007; Sugar et al., 2011). Both the granular and dysgranular retrosplenial cortices densely innervate the postsubiculum and presubiculum, as well as projecting to the medial and lateral entorhinal cortices (Jones and Witter, 2007). These connections involve the cingulum. There are also a few direct retrosplenial projections to the subiculum, which arise from Rgb (Sugar et al., 2011). In contrast, the anterior cingulate cortex has more restricted projections, which terminate in the perirhinal cortex and lateral entorhinal cortex (Jones and Witter, 2007). Some of these anterior cingulate efferents do not join the cingulum (Jones and Witter, 2007).

**Figure 5.**
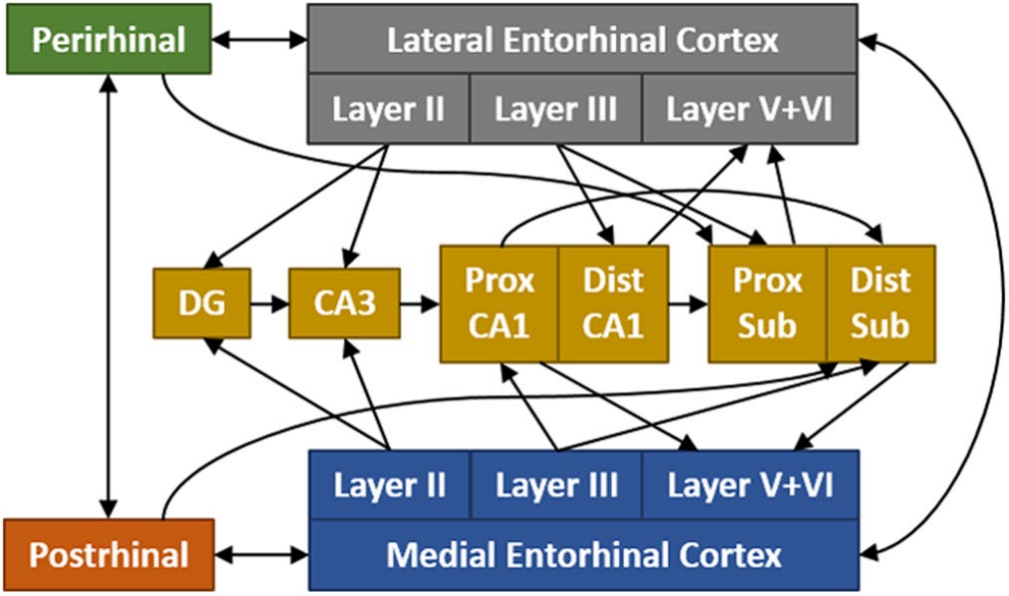
Schematic showing the major hippocampal–parahippocampal interactions in the rat brain. These interconnections are organised by both their proximal–distal locations and the lamina of their inputs and outputs. CA: CA fields of the hippocampus; Dist: distal; DG: dentate gyrus; Prox: proximal; Sub: subiculum. (For simplicity, the presubiculum and parasubiculum are not included.)

The presubiculum and postsubiculum have dense projections to the entorhinal cortices (Van Groen and Wyss, 1990b, 1990c), thereby completing an additional pathway back to the hippocampus. Entorhinal projections terminate in either the dentate gyrus and CA3 (via the perforant pathway) or CA1 and the subiculum (via the temporoammonic pathway). These connections (Figure 5) are largely segregated by the lamina of their origin within the entorhinal cortex (layer II to the dentate gyrus and CA3, layer III to CA1 and subiculum). Many reviews have detailed the numerous parahippocampal–hippocampal interconnections (see Furtak et al., 2007; Van Strien et al., 2009).

### Completing the hippocampal–diencephalic–cingulate network

**Figure 6.**
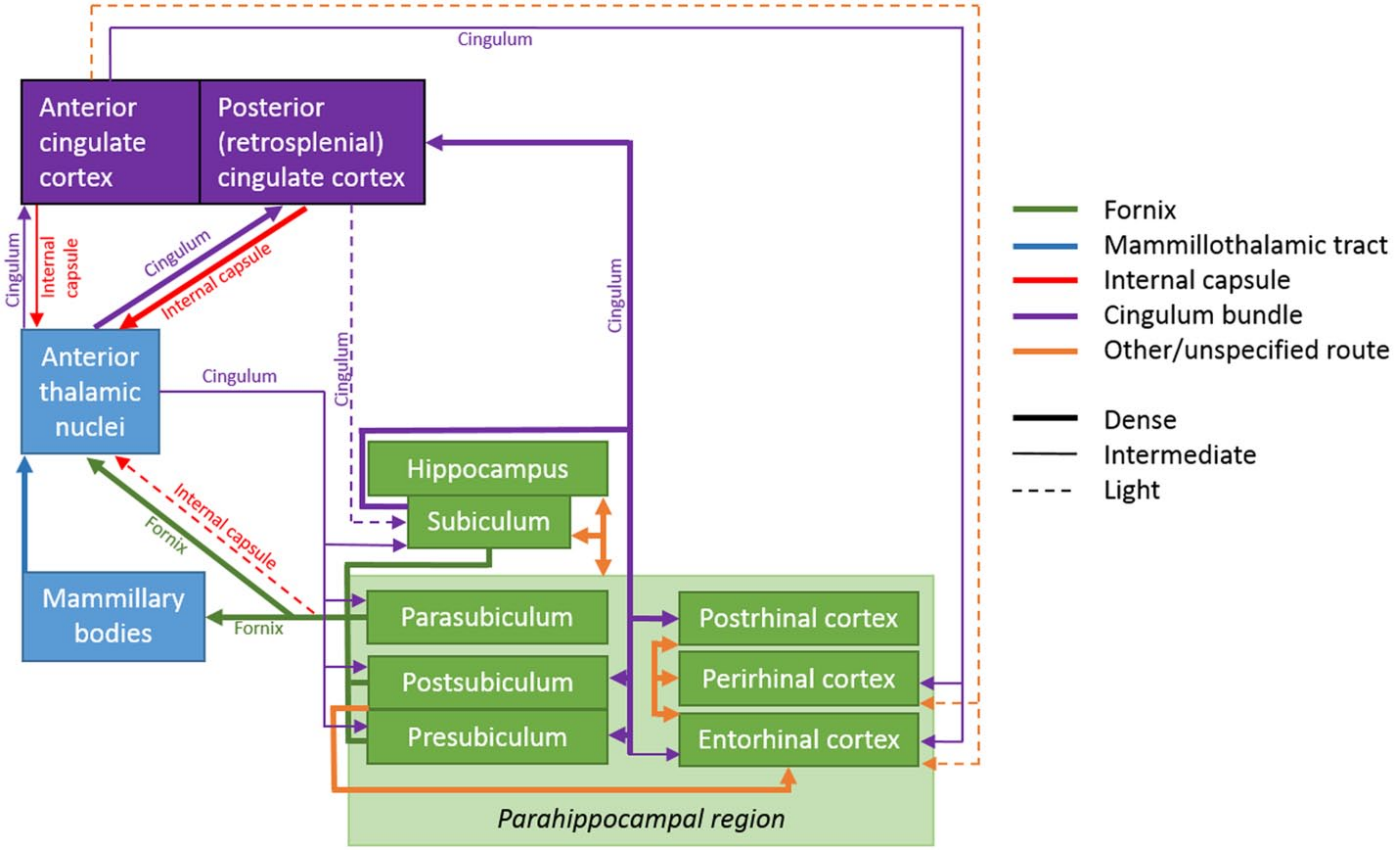
The rat brain: Schematic showing the main, direct interconnections between sites in Papez circuit. The style of the lines reflects the strength of the connections (thick line = dense, thin line = intermediate, dashed line = light).

While the core connections described by Papez (Figure 1) exist in the rat, many other connections add to its complexity. Some projections seemingly bypass one or more stages (Figures 3–6). A striking example, already described, concerns the dense, direct projections from the subiculum to the anterior thalamic nuclei. Other examples include the direct projections from the anterior thalamic nuclei to caudal hippocampal (subiculum) and parahippocampal areas. Dense projections from both the anteroventral and anterodorsal thalamic nuclei focus on the presubiculum, parasubiculum, and postsubiculum, with the anteroventral nucleus also innervating the caudal subiculum (Shibata, 1993). These projections are thought to take a very similar route, passing forward and then upwards from the thalamus to join the cingulum, before travelling caudally, while some of those from the anterodorsal nucleus may take a direct route that does not involve the cingulum (Shibata, 1993; Shibata and Kato, 1993; Van Groen and Wyss, 1990b, 1990c). Meanwhile, the anteromedial nucleus has light projections to the ventral subiculum, but more appreciable projections to the perirhinal and entorhinal (medial and lateral) cortices (Shibata, 1993). The anterodorsal thalamic nucleus also projects to the entorhinal cortices (Shibata, 1993). Other connections include efferents from the medial entorhinal cortex to the anteroventral thalamic nucleus (Shibata, 1996), which involve the internal capsule, that is, they take a nonfornical route (Figure 3).

As already noted, there are connections that project in the opposite direction to that portrayed in depictions of Papez’s original circuit. One example, already discussed, concerns the dense projections from the cingulate cortices to the anterior thalamic nuclei (Figures 4 and 6). The significance of these projections is highlighted by a viral tracing study showing that a major pathway from the retrosplenial cortex to the dorsal hippocampus is via the anterior thalamic nuclei (Prasad and Chudasama, 2013). There are also many projections from the subiculum and parahippocampal regions to the retrosplenial cortices. It is principally the distal subiculum that projects directly to area 29 (Rga and Rgb; Honda and Ishizuka, 2015), where fibres terminate in layers I, II, and III. The postsubiculum also projects to Rgb, with terminations in layers I and III–V (Van Groen and Wyss, 2003). There are also light projections from CA1 to area 29 (Jones and Witter, 2007). Finally, there is a very light, direct pathway from the granular retrosplenial cortex to the medial mammillary bodies, which appears to join the postcommissural fornix (Van Groen and Wyss, 2003).

Figure 6 depicts a simplified, but updated, hippocampal–diencephalic–cingulate network for the rat. Arguably, the most striking feature is how almost all the structures project to more than one site within the network. The mammillary bodies provide the sole exception, as they only project to the anterior thalamic nuclei (Figure 6). An integral feature is how the various connections within the circuit are topographically organised, creating parallel pathways that presumably reflect multiple functions (Aggleton et al., 2010; Vann and Aggleton, 2002; Vertes et al., 2004).

## The monkey hippocampal–diencephalic–cingulate network

### Hippocampus to mammillary bodies

**Figure 7.**
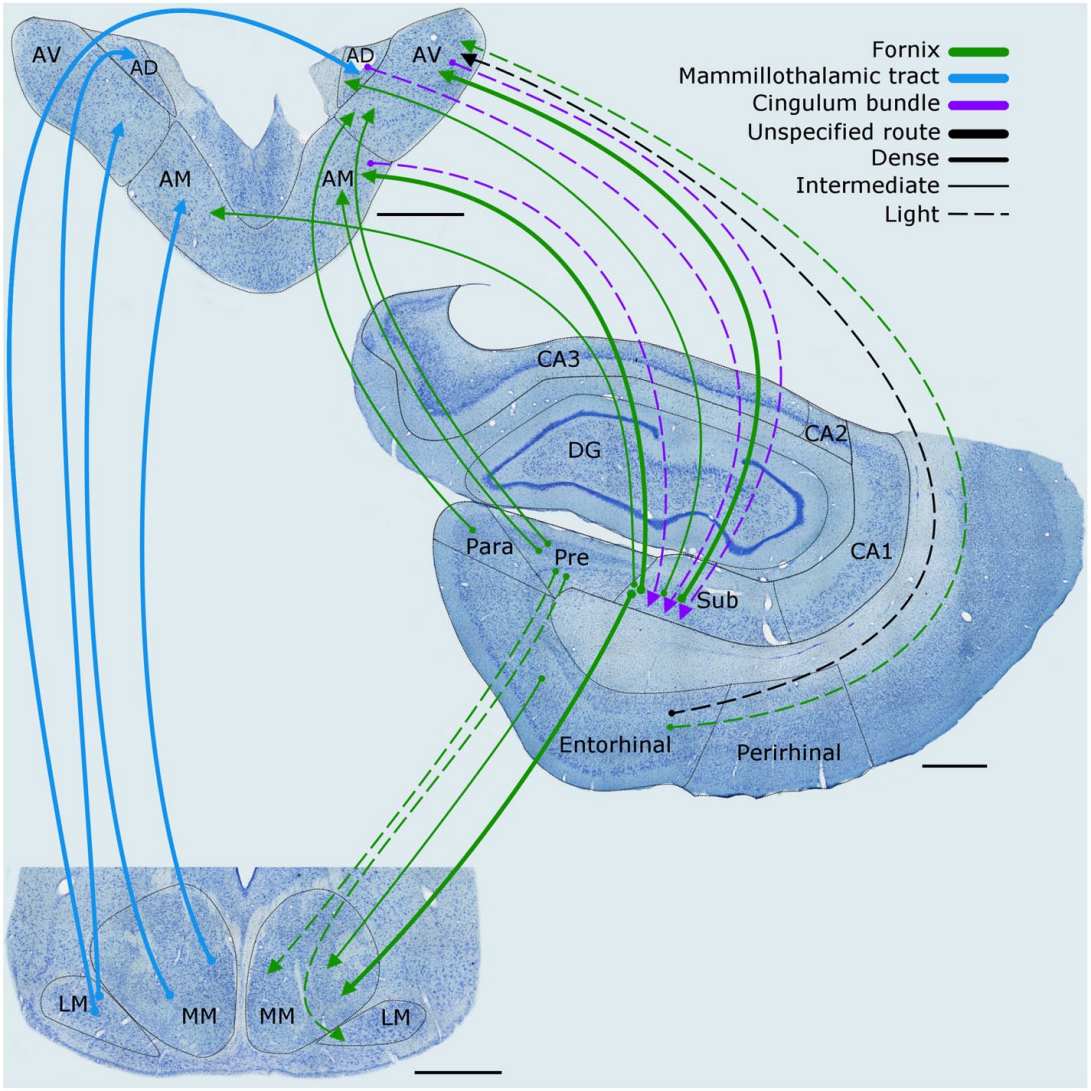
Macaque monkey brain. Depiction of the connections between the hippocampal and parahippocampal regions with the mammillary bodies and anterior thalamic nuclei, as well as the projections from the mammillary bodies to the anterior thalamic nuclei. The routes of these connections are distinguished by different colours. The origin of a connection is denoted by a circle and the termination is signified by an arrowhead while a reciprocal connection that follows the same route has an arrowhead at both ends. The style of the lines reflects the strength of the connections (thick line = dense, thin line = intermediate, dashed line = light). The upper left panel shows the anterior thalamic nuclei, while the lower left panel depicts the mammillary bodies. AD: anterodorsal nucleus; AM: anteromedial nucleus; AV: anteroventral nucleus; CA1–3: CA fields of the hippocampus; DG: dentate gyrus; LM: lateral nucleus of the mammillary bodies; MM: medial nucleus of the mammillary bodies; MML: lateral division of the medial mammillary nucleus; MMM: medial division of the medial mammillary nucleus; Para: parasubiculum; Pre: presubiculum; Sub: subiculum. Scale bars = 1000 µm.

**Figure 8.**
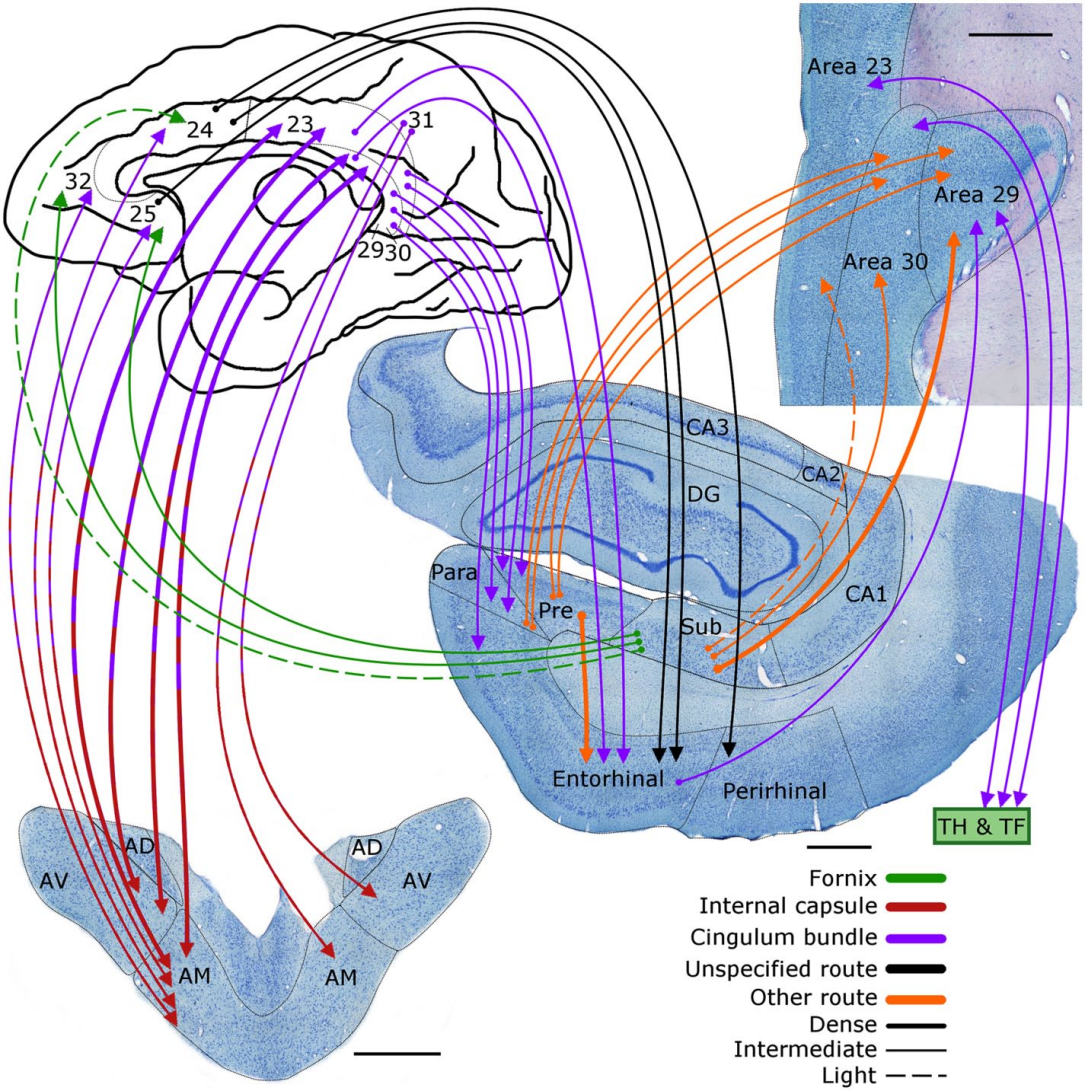
Macaque monkey brain. Depiction of the connections between the anterior thalamic nuclei (lower left), cingulate gyrus (areas 23, 24, 25, 29, 30, 31, 32), hippocampus, and parahippocampal regions. The routes of these projections are distinguished by different colours. In the case of some connections, two colours are used to show how they pass from other pathway to another. The origin of a connection is denoted by a circle and the termination is signified by an arrowhead while a reciprocal connection that follows the same route has an arrowhead at both ends. AD: anterodorsal nucleus; AM: anteromedial nucleus; AV: anteroventral nucleus; CA1–3: CA fields of the hippocampus; DG: dentate gyrus; Para: parasubiculum; Pre: presubiculum; Sub: subiculum. Scale bars = 1000 µm.

The network connections in macaque monkeys (*Macaca mulatta and Macaca fascicularis*) are very similar to those described for the rat brain (Figures 7 and 8). Once again, the hippocampal projections to the mammillary bodies originate from the subiculum (Aggleton et al., 2005). These subicular projections (Figure 7), which arise from pyramidal cells, join the body of the fornix and then descend in the postcommissural fornix, where they make up approximately one half of its fibres (Powell et al., 1957). The neurons projecting to the medial mammillary nucleus are most numerous in the distal and posterior subiculum (Christiansen et al., 2016b). As in the rat, there is evidence of a horizontal topography in their terminations, as the anterior subiculum projects to more ventral aspects of the medial mammillary nucleus while the posterior subiculum projects more dorsally (Aggleton et al., 2005). In addition, light projections from the presubiculum reach the medial and lateral mammillary nuclei, while the entorhinal cortex projects to the medial mammillary nucleus (Aggleton et al., 2005).

### Mammillary bodies to the anterior thalamic nuclei

The very dense mammillary body projections to the anterior thalamus are organised such that neurons in the dorsal medial mammillary nucleus project to the anteromedial thalamic nucleus while the remainder of the medial mammillary nucleus projects to the anteroventral thalamic nucleus (Vann et al., 2007; Xiao and Barbas, 2002). The lateral mammillary nucleus projects to the anterodorsal thalamic nucleus, although it may also provide light inputs to other anterior thalamic nuclei (Vann et al., 2007). While the lateral mammillary inputs to the anterodorsal nucleus are bilateral, the medial mammillary projections to the other anterior thalamic nuclei remain ipsilateral. It is again thought that almost every neuron in the primate mammillary bodies contributes to the anterior thalamic projections (Powell et al., 1957; Xiao and Barbas, 2002).

As in the rat, the indirect route from the hippocampus to the anterior thalamic nuclei via the mammillary bodies is reinforced by numerous, direct projections from the hippocampus to the anteromedial and anteroventral thalamic nuclei, with lighter inputs to the anterodorsal nucleus (Figure 7). These hippocampal projections arise from the deepest cell layer of the subiculum, ensuring that they are segregated from the hippocampal projections to the mammillary bodies, which are found in the middle layer (Aggleton et al., 1986; Christiansen et al., 2016b). The projections to the anterior thalamus predominantly arise from the distal subiculum before joining the fornix (Christiansen et al., 2016b). While the hippocampal inputs to the anteromedial thalamic nucleus are bilateral, those to the anteroventral nucleus and the anterodorsal nucleus essentially remain ipsilateral (Aggleton et al., 1986). The presubiculum provides light inputs to the anteroventral and anteromedial thalamic nuclei, with even lighter inputs arising from the parasubiculum (Saunders et al., 2005; Xiao and Barbas, 2002). In addition, there are light projections from the entorhinal cortex and perirhinal cortex to the anterior thalamic nuclei (especially to the anteroventral nucleus), some of which join the fornix (Saunders et al., 2005; Xiao and Barbas, 2002).

### Anterior thalamic nuclei to cingulate gyrus

The dense anterior thalamic projections to the cingulate cortices (Figure 8) are most concentrated in the posterior cingulate region (Baleydier and Mauguiere, 1980; Shibata and Yukie, 2009; Vogt et al., 1979, 1987). This region in the macaque comprises posterior cingulate areas 23 and 31, as well as retrosplenial areas 29 and 30. Both the anteroventral and anteromedial nuclei project to ventral area 23, with lighter projections to dorsal area 23 (Morris et al., 1999; Shibata and Yukie, 2009; Vogt et al., 1987). These projections to area 23 principally arise from the anteromedial nucleus (Vogt et al., 1987). Meanwhile, the anteroventral nucleus provides most of the projections to area 30, while those to area 29 are from both the anteroventral and anterodorsal thalamic nuclei (Shibata and Yukie, 2009; Vogt et al., 1987).

There are dense, return projections from layer VI of the posterior cingulate region to the anterior thalamic nuclei (Aggleton et al., 2014). Area 23 projects to both the anteromedial and anteroventral thalamic nuclei, alongside lighter projections from area 31 to the same thalamic nuclei (Aggleton et al., 2014; Shibata and Yukie, 2009). The retrosplenial efferents from areas 29 and 30 principally target the anteroventral thalamic nucleus, with lighter inputs to the anteromedial nucleus. It is presumed, but not certain, that area 29 also projects to the anterodorsal thalamic nucleus (Shibata and Yukie, 2009). The inputs to the anteromedial nucleus from areas from 23 and 30 include crossed connections from the other hemisphere (Aggleton et al., 2014; Shibata and Yukie, 2009).

The anterior thalamic–posterior cingulate connections have a reciprocal organisation (Figure 8). While areas 29 and 30 especially interact with the anteroventral nucleus, the anteromedial nucleus is especially connected with area 23 (and areas 24, 25, and 32). Many of the anterior thalamic projections to the posterior cingulate region leave the thalamus laterally, before passing around the caudate nucleus in the internal capsule to join and cross the cingulum. The reciprocal projections from the posterior cingulate region to the thalamus appear to take essentially the same route (Mufson and Pandya, 1984).

The anterior cingulate cortex receives fewer inputs from the anterior thalamic nuclei than its posterior counterpart (Vertes et al., 2015; Vogt et al., 1987). Area 24, which forms much of this region, receives light inputs from the anteromedial nucleus (Baleydier and Mauguiere, 1980; Shibata and Yukie, 2009; Vogt et al., 1987). The same thalamic nucleus also gives rise to modest projections to medial frontal areas 32 and 25, which are also part of the anterior cingulate region (Shibata and Yukie, 2009; Vogt et al., 1987). These thalamic projections to anterior areas 24, 25, and 32 leave the thalamus rostrally to pass the anterior limb of the internal capsule before joining and crossing the cingulum (Mufson and Pandya, 1984). Other anterior thalamic projections to area 24 cross the dorsal thalamus to skirt around the caudate nucleus before turning medially to join and cross the cingulum. The projections to areas 25 and 32 are of additional note as these same areas receive many of the direct hippocampal (CA1, subiculum) projections to prefrontal cortex (Aggleton et al., 2015).

There are also return projections from the anterior cingulate region to the anterior thalamic nuclei (Figure 8). While the densest thalamic projections from area 24 terminate in the medial dorsal thalamic nucleus, there are some projections to the anteromedial nucleus (Baleydier and Mauguiere, 1980; Shibata and Yukie, 2009). Likewise, areas 25 and 32 project to the anteromedial thalamic nucleus (Xiao and Barbas, 2002), but for all three of these anterior cingulate areas (24, 25, and 32) it is the reciprocal connections with the medial dorsal thalamic nucleus that are the most dense (Shibata and Yukie, 2009).

### Cingulate cortex to the parahippocampal region and hippocampus

Numerous inputs arise from across retrosplenial areas 29 and 30, as well as ventral 23 in the posterior cingulate region, to reach the parahippocampal region (Yukie and Shibata, 2009). Many of these projections terminate in the presubiculum and parasubiculum, with relatively few fibres innervating the subiculum (Kobayashi and Amaral, 2007; Morris et al., 1999). The presubiculum projects to the entorhinal cortex, thereby completing the circuit (Figure 8). In addition, there are dense, direct projections from the retrosplenial cortex and ventral area 23 to entorhinal cortex, as well as to areas TH and TF of the parahippocampal region (Insausti et al., 1987a; Kobayashi and Amaral, 2007; Pandya et al., 1981; Suzuki and Amaral, 1994). Dorsal area 23 has more restricted projections, focussed on area TF (Kobayashi and Amaral, 2007).

The anterior cingulate region (areas 24, 25, and 32) has light, reciprocal connections with much of the parahippocampal region (Insausti et al., 1987a; Pandya et al., 1981; Suzuki and Amaral, 1994; Yukie and Shibata, 2009). There are, for example, light interconnections between the perirhinal cortex and ventral area 24, as well as with area 32. In addition, both the entorhinal cortex and areas TH/TF have modest, reciprocal connections (not depicted in figures) with areas 24, 25, and 32 (Yukie and Shibata, 2009).

### Completing the hippocampal–diencephalic–cingulate network

**Figure 9.**
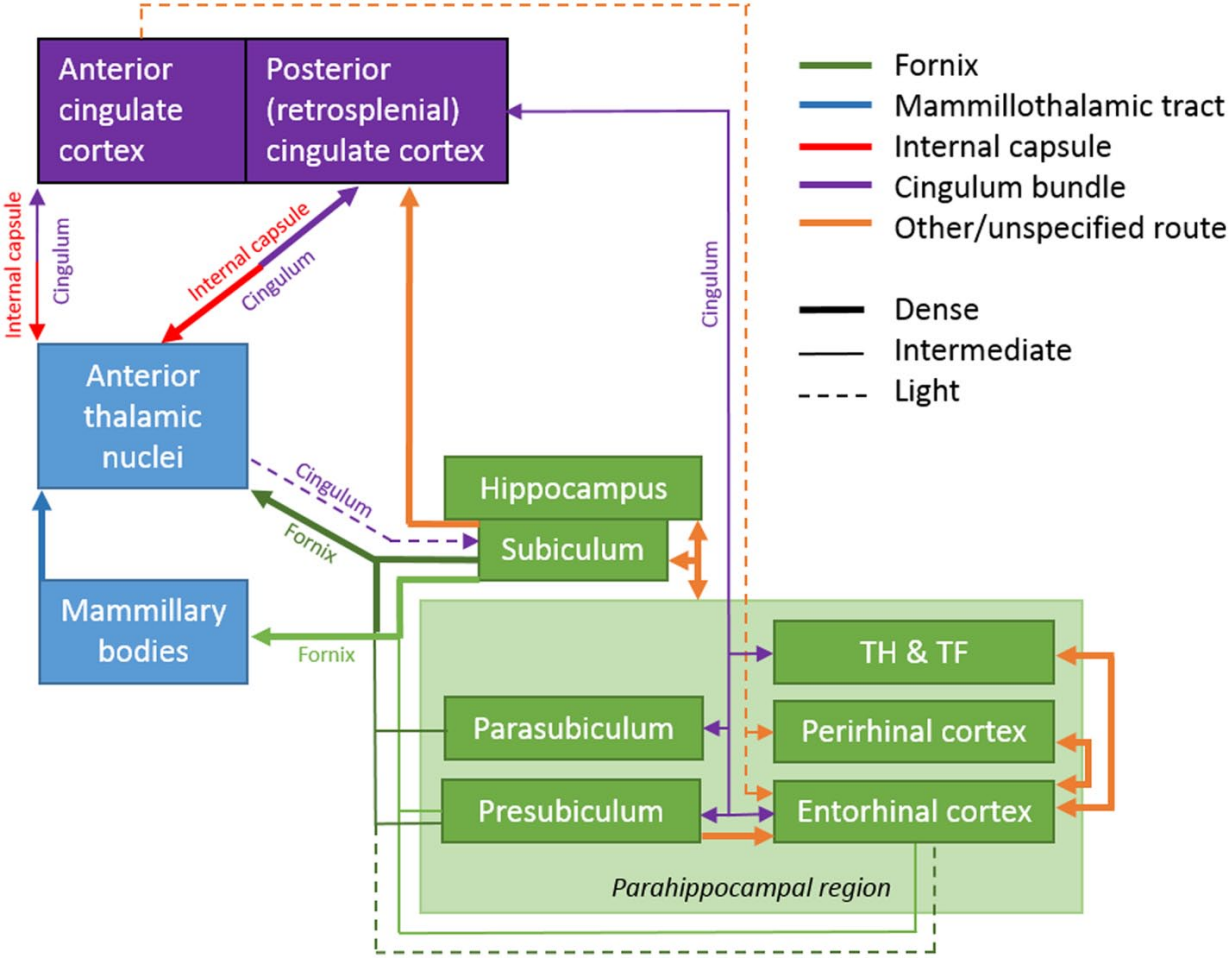
Schematic showing the major interconnections of the hippocampal–diencephalic–cingulate network in the macaque monkey. In the case of some connections, two colours are used to show how they pass from one pathway to another. The style of the lines reflects the strength of the connections (thick line = dense, thin line = intermediate, dashed line = light).

To help clarify the situation, Figure 9 depicts a simplified version of the updated hippocampal–diencephalic–cingulate network, highlighting its principal connections. Based on the pattern of thalamic connections, it can be seen that within the cingulate gyrus, the retrosplenial cortex forms a particularly important link in this limbic subsystem (Figures 8 and 9). Consistent with this distinction, the direct projections from the hippocampus to the cingulate gyrus are largely restricted to the retrosplenial cortex. Dense inputs to the retrosplenial cortex arise from the subiculum, as well as from the presubiculum, and parasubiculum, (Aggleton et al., 2015; Kobayashi and Amaral, 2003; Morris et al., 1999; Parvizi et al., 2006; Vogt et al., 1987). The subiculum preferentially innervates area 29 (layers I and III), with less dense projections reaching area 30 (layer III; Aggleton et al., 2012; Kobayashi and Amaral, 2003). The anterior subiculum targets more ventral retrosplenial areas while the posterior subiculum has denser projections to mid and dorsal retrosplenial cortex, as well as a light input to area 23 (layer III). This pattern is matched by the return projections from retrosplenial cortex, for example, the most ventral parts of 29/30 project to the anterior hippocampus (presubiculum; Kobayashi and Amaral, 2007). In contrast, there are only very limited subiculum inputs to area 24 in the anterior cingulate region, although there are more inputs from the subiculum to areas 25 and 32, which reach these sites via the fornix (Aggleton et al., 2015).

The retrosplenial cortex also receives direct inputs from the parahippocampal region. Areas TH and TF provide appreciable projections to areas 29 and 30, with lighter efferents to area 23 (Kobayashi and Amaral, 2003; Lavenex et al., 2002; Morris et al., 1999). Additional retrosplenial inputs arise from the caudal entorhinal cortex, which focus on area 29 (Aggleton et al., 2012; Kobayashi and Amaral 2003; Morris et al., 1999). A feature of the subiculum projections to the retrosplenial cortex is that they do not join the cingulum, rather they cross directly through the presubiculum to reach the posterior cingulate cortices directly (Aggleton et al., 2012).

As in other species, the anterior thalamic nuclei have direct projections to the hippocampal formation (Amaral and Cowan, 1980). These light thalamic projections, which join the caudal cingulum (Mufson and Pandya, 1984), arise from all three major anterior thalamic nuclei and terminate in the region of the subiculum (Amaral and Cowan, 1980). The anterior thalamic nuclei do not, however, appear to project directly to the entorhinal cortex (Insausti et al., 1987b).

### Monkey versus rat

Although the core connections in the monkey hippocampal–diencephalic–cingulate network are, in many respects, very similar to those in rodents, there are some differences. Unsurprisingly, most of these differences reflect the connections of the cingulate cortices. These changes partly arise from the additional areas present in the primate brain (areas 23 and 31), while other areas (25 and 32) do not have precise counterparts in the rat brain (Vogt and Paxinos, 2014). Other challenges arise from the frequent failure to distinguish a midcingulate cortex in rodents which is evident in primates (Vogt, 2009) and can be identified in rodents (Vogt and Paxinos, 2014). Nevertheless, in both rats and macaque monkeys, it is the retrosplenial cortex that forms the principal cingulate node within the hippocampal limbic network.

In the rat brain, there is a clear cingulate–thalamic demarcation as the retrosplenial cortex (areas 29 and 30) is interconnected with the anterior thalamic nuclei but not with the medial dorsal thalamic nucleus (Van Groen and Wyss, 1990a, 1992, 2003). In the monkey, this same distinction remains but is not so marked, as the retrosplenial cortex now has light interconnections with the medial dorsal thalamic nucleus (Aggleton et al., 2014; Vogt et al., 1987). There are also additional connections involving the monkey posterior cingulate region, area 23, which is more clearly connected with both the anterior thalamic nuclei and the medial dorsal nucleus (Aggleton et al., 2014; Shibata and Yukie, 2009; Vogt et al., 1987). Likewise, the anterior cingulate cortex is interconnected with both the anterior thalamic nuclei and the medial dorsal nucleus in rats and macaque monkeys (Shibata, 1993; Shibata and Naito, 2005; Shibata and Yukie, 2009). Furthermore, in both species, these anterior cingulate connections are focussed on the anteromedial thalamic nucleus.

One species difference involves the topographic organisation of the subiculum neurons that project to the mammillary bodies and the anterior thalamic nuclei (Christiansen et al., 2016b). In the rat, this organisation is partly based on the proximal–distal plane, but in the monkey this separation is achieved by laminar position only. Finally, it might be supposed that the emergence of additional prefrontal connections would create very different network relationships across species. In fact, this difference is not so marked as may be imagined as the rat prelimbic cortex has many corresponding connections with the hippocampal–diencephalic–cingulate network as those seen with primate medial frontal areas, for example, with the anteromedial thalamic nucleus and with the hippocampus (CA1 and subiculum).

## The human hippocampal–diencephalic–cingulate network

The inability to use axonal tract tracers means that our knowledge of this system in the human brain remains superficial. One approach has been to use fibre dissection techniques. In this way, the tracts from the hippocampus to the mammillary bodies (postcommissural fornix), from the mammillary bodies to the anterior thalamus (mammillothalamic tract), from the anterior thalamus to the cingulate cortices (anterior thalamic radiations and cingulum), and, finally, from the cingulate cortices to the parahippocampal region (posterior cingulum) can be visualised (Shah et al., 2012). One interesting finding is that the majority of fibres in the body of the fornix join the postcommissural fornix (Shah et al., 2012), emphasising the likely importance of the connections highlighted by Papez, as the postcommissural pathway principally terminates in the mammillary bodies and the anterior thalamic nuclei. The fibre dissection technique is, however, limited as it cannot confirm the direction of a set of fibres and fails to reveal diffuse pathways.

Some of the same limitations apply to magnetic resonance imaging (MRI) techniques such as diffusion tensor imaging (DTI) and diffusion spectrum imaging (DSI). Nevertheless, with these techniques, it is possible to reconstruct pathways such as the fornix (including the postcommissural fornix), the mammillothalamic tract, and the cingulum (Catani et al., 2013; Christiansen et al., 2016a; Granziera et al., 2011; Jones et al., 2013; Kwon et al., 2010; Wei et al., 2017), with the added ability to look for changes in axonal properties based on pathology or experience (see section ‘Anatomy and function’). It has also been possible to show that as in macaques, the retrosplenial cortex is the main cingulate site for direct hippocampal connections (Wei et al., 2017). With the exception of the mammillothalamic tract, there is, however, the added problem for these imaging methods (and microdissection) that all these tracts connect multiple sites, that is, their fibres are not restricted to the hippocampal limbic network. For this reason, the mammillothalamic tract is the only pathway devoted to Papez’s part of the limbic system.

## Re-connecting the hippocampal–diencephalic–cingulate network

From Figures 3 to 9, it is immediately evident that the connectivity in these pathways is more complex than often described. While we cannot be certain of the fine details in the human brain, a great many features are shared in both the rat and macaque brain, suggesting that they are also present in our brains. One of these common features, the bypassing of stages within the serial circuit described by Papez (e.g. the direct hippocampal projections to the anterior thalamic nuclei), reinforces the concept of an integrated system as it strengthens the close interactions between serial sites. This concept is further strengthened by the many reciprocal connections within the network. The many bypassing connections do, however, pose questions about the computational value of combined direct and indirect projections between the same sites, a feature that occurs repeatedly within this limbic subsystem. This feature is arguably most striking in the anterior thalamic nuclei, which receive dense direct hippocampal inputs alongside dense, indirect inputs (hippocampus → mammillary body → anterior thalamic nuclei; hippocampus → retrosplenial cortex → anterior thalamic nuclei).

A further feature of the network is the presence of clear topographies within every pathway. These topographies imply parallel functions set within the same broad set of connections (Aggleton et al., 2010). One well-established example concerns the head-direction system, which aids navigation (Taube, 2007). In the rodent, cells signalling this direction information are especially prevalent in the lateral mammillary nucleus, the anterodorsal thalamic nucleus, the lateral dorsal thalamic nucleus, the retrosplenial cortex, and the postsubiculum. Consequently, there is a head-direction subsystem set within Papez’s original circuit (Vann and Aggleton, 2004). Other functional divisions are presumably reflected by the topographic differences in the connections of the anteromedial and anteroventral thalamic nuclei (Figures 3 and 4; Aggleton et al., 2010). One example concerns the relative switch in influence between thalamic–frontal interactions (anteromedial nucleus) and thalamic–hippocampal interactions (anteroventral nucleus). It should be added that these topographic divisions extend to subregions within a nucleus (Shibata, 1992; Shibata and Kato, 1993).

A potentially important issue concerns the distinction between the anterior cingulate and posterior cingulate regions. These two regions differ in the strength and breadth of interconnections with the anterior thalamic nuclei, the hippocampus, and parahippocampal region (Kobayashi and Amaral, 2003, 2007; Shibata and Yukie, 2009; Vertes et al., 2015; Yukie and Shibata, 2009) making the posterior cingulate region (especially retrosplenial cortex) much more closely tied to the hippocampal–diencephalic–cingulate network. In contrast, the much greater levels of interaction between the anterior cingulate region and the amygdala indicate that this cortical area is better seen as part of a different limbic subsystem more involved in emotion (Catani et al., 2013; Dalgleish, 2004; Ranganath and Ritchey, 2012; Rolls, 2015). This different system has been called the ‘basolateral limbic system’ (Livingston and Escobar, 1971). It is, however, important to appreciate that the anterior cingulate–posterior cingulate distinction is not absolute as both regions are reciprocally interconnected and they are both connected with the anterior thalamic nuclei and the mediodorsal thalamic nucleus (Baleydier and Mauguiere, 1980). Consequently, the cingulate cortices have a potentially important role in providing cross-talk between these two major limbic subsystems (Baleydier and Mauguiere, 1980; Livingston and Escobar, 1971; Rolls, 2015). In addition, the hippocampus, entorhinal cortex, and amygdala are all reciprocally connected (Aggleton, 1986; Saunders et al., 1988) providing further interplay between these putative systems.

## Anatomy and function

**Figure 10.**
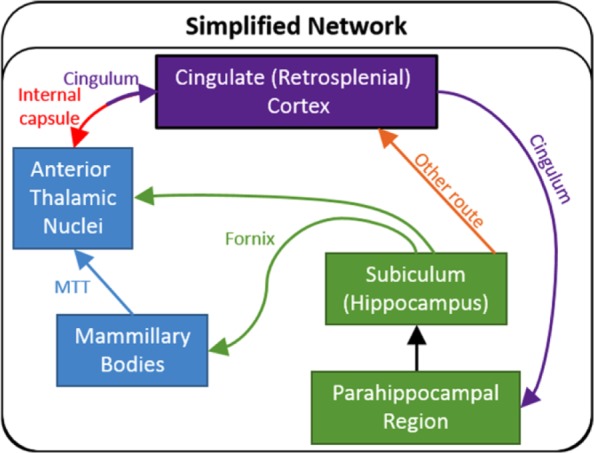
Simplified schematic showing the major common connections in both the rat and monkey (macaque) brains between those sites comprising the hippocampal–diencephalic–cingulate network. When updated, it is apparent that Papez ‘circuit’ can also be interpreted as twin routes (dorsal and ventral) from the hippocampal and parahippocampal regions to the cingulate cortices and thalamus. The twin colour of the pathway between the anterior thalamic nuclei and the cingulate cortex reflects the involvement of two tracts. MTT: mammillothalamic tract.

For both historic reasons and to reflect current research priorities, there has been a natural tendency to emphasise the hippocampus within the hippocampal–diencephalic–cingulate network. This emphasis has been reinforced by the realisation that the network’s connections are required for memory (see below). For this same reason, the hippocampus is still often seen as both the principal start and finish points for many of the connections within this limbic subsystem (Aggleton and Brown, 1999; Rolls, 2015). An unfortunate consequence of this viewpoint is that it tends to diminish the perceived importance of the individual steps around the ‘circuit’ beyond the hippocampus, as a return loop implies that these additional stages are not always needed. In fact, this hippocampal focus, while understandable, has no particular anatomical claim. Indeed, an alternate way to consider this network is to regard the connections as principally a set of direct and indirect projections *from* the medial temporal lobe *to* the anterior thalamus and cingulate cortices, one function of which will be to engage additional cingulate and prefrontal areas. Consequently, the connections might better be considered as parallel projections emanating from hippocampal and parahippocampal regions, creating a medial temporal lobe efferent system, rather than a circuit (Figure 10), despite the many return connections. When re-cast in this way, it is striking how many of the connections within this limbic subsystem are also components of the ‘default mode network’ (Greicius et al., 2003; Raichle, 2015).

This review helps explain one reason why it has proved difficult to appreciate the contributions of structures beyond the hippocampus in this network. The difficulty arises from the complexity of their connections. The presence of parallel, bypassing projections (Figures 6 and 9) will typically protect against the full effects of damage to an individual site beyond the hippocampus, as only incomplete disconnections can occur. One apparent exception is provided by the anterior thalamic nuclei, which receive convergent inputs from the hippocampus, mammillary bodies, and cingulate cortices. While the lack of clinical conditions that selectively target the anterior thalamic nuclei makes it difficult to test this prediction in humans (but see Harding et al., 2000), support comes from lesion studies in rats. Such experiments have repeatedly confirmed that these thalamic nuclei are vital for hippocampal-dependent learning (Henry et al., 2004; Warburton et al., 2001). Furthermore, anterior thalamic lesions are typically more disruptive than the corresponding lesions in the mammillary bodies (Aggleton et al., 1991, 1995a) and can, sometimes, be more disruptive than fornix lesions (Warburton and Aggleton, 1998). These findings presumably reflect the array of direct and indirect connections within this network that converge on the anterior thalamic nuclei. For opposite reasons, it is supposed that the impact of retrosplenial cortex lesions in rats can often be mitigated by the many parallel hippocampal pathways that can bypass this area (Nelson et al., 2015).

### Memory and amnesia

The current concepts on the nature of the hippocampal–diencephalic–cingulate network are strongly affected by the realisation that its interconnections are vital for normal episodic memory (Aggleton and Brown, 1999; Catani et al., 2013; Rolls, 2015). The switch from emotion (Papez, 1937) to memory followed the description of temporal lobe amnesic cases, including patient, H.M. (Delay and Brion, 1969; Scoville and Milner, 1957; Spiers et al., 2001). Complementary evidence came from neuropathological studies of the amnesic Korsakoff’s syndrome, which has repeatedly implicated the mammillary bodies and, thereby, the anterior thalamic nuclei in diencephalic amnesia (Delay and Brion, 1969; Harding et al., 2000; Kopelman, 1995; Victor et al., 1989). Further support comes from studies of amnesic patients with thalamic vascular accidents and those with colloid cysts in the third ventricle (Carlesimo et al., 2011; Tsivilis et al., 2008; Van der Werf et al., 2003). Critically, it has also been shown that fornix damage is sufficient to cause anterograde amnesia (Aggleton et al., 2000; Gaffan et al., 1991; Gaffan and Gaffan, 1991; McMackin et al., 1995), thereby linking together hippocampal and medial diencephalic sites for episodic memory (Aggleton et al., 2000; Rudebeck et al., 2009; Tsivilis et al., 2008; Vann et al., 2009b).

Combining these neuropsychological findings led to the proposal that all the sites and connections interlinking the hippocampal formation with the medial diencephalon and posterior cingulate cortices work together to support memory in an ‘extended hippocampal system’ (Aggleton and Brown, 1999, 2006). An apparent problem, however, is that cingulum damage does not appear to be sufficient to induce amnesia in humans (Ballantine et al., 1987; Feldman et al., 2001; Turner, 1973), despite the considerable involvement of this tract for connections between the cingulate cortices and the parahippocampal region (and, hence, the hippocampus). Likewise, posterior cingulum bundle lesions in rats often have only mild effects on spatial memory tasks that are highly sensitive to hippocampal damage (Aggleton et al., 1995b; Neave et al., 1996). While techniques such as DTI have helped to reveal the likely importance of the cingulum bundle for some aspects of cognition, including cognitive control, there remains a failure to find memory functions that resemble those of the fornix (Hollocks et al., 2015; Metzler-Baddeley et al., 2012; Turner, 1973; see also Aggleton et al., 1995b; Neave et al., 1996).

Part of the explanation for these negative findings is anatomical. The idea of a hippocampal-based return circuit ignores how the connections in this limbic subsystem are reciprocal. Furthermore, as described above, the dense hippocampal (subiculum) projections to retrosplenial cortex do not join the cingulum (Aggleton et al., 2012). For these reasons, it is less likely that cingulum bundle damage would be sufficient to induce amnesia. That said, there is much evidence that the retrosplenial cortex is important for multiple aspects of memory (Maguire, 2001; Vann et al., 2009a), including the existence of ‘retrosplenial amnesia’ (Valenstein et al., 1987). This account implies that many retrosplenial connections important for memory do not rely on the cingulum, examples of which include the projections from the hippocampus to retrosplenial cortex and some of those between the retrosplenial cortex and the anterior thalamic nuclei (Aggleton et al., 2012, 2014; Mufson and Pandya, 1984). Another part of the explanation is conceptual. As already noted, there is an understandable tendency to see the hippocampus as the centre of a circuit around which information flows, but with little alteration in the information itself. This misconception leads to the false idea that cingulum bundle damage and fornix damage will have similar effects on memory, notwithstanding the fact that both pathways also contain numerous fibres involved in other connections.

Despite these limitations, the realisation that individual components of the hippocampal–diencephalic–cingulate network have key roles in episodic memory is proving increasingly insightful when trying to understand the functional neuropathology of disorders such as amnesic mild cognitive impairment and Alzheimer’s disease (Aggleton et al., 2016; Nestor et al., 2003; Tan et al., 2013; Vogt et al., 2009). Over recent decades, it has often been assumed that hippocampal and parahippocampal dysfunctions are responsible for the memory loss in these neurological conditions. In fact, there is growing evidence that structures in Papez’s original circuit beyond the parahippocampal and hippocampal regions also show pathological changes and activity abnormalities during the prodromal stages of these same disorders (Aggleton et al., 2016; Minoshima et al., 1997; Nestor et al., 2003). Consequently, to understand the origins of the memory loss in conditions such as mild cognitive impairment and Alzheimer’s disease, it will be necessary to broaden our perspectives to incorporate anterior thalamic and retrosplenial sites (Aggleton et al., 2016; Hornberger et al., 2012). An important aspect of this realignment is the growing realisation that sites such as the mammillary bodies and anterior thalamic nuclei make contributions to learning and memory that are not solely dependent on their hippocampal inputs (Dillingham et al., 2015b; Wright et al., 2015).

Unlike other structures within the hippocampal–diencephalic–cingulate network, damage to the anterior cingulate region does not produce an anterograde amnesia, despite recording data revealing long-term mnemonic functions (Xiang and Brown, 2004). Instead, there is considerable imaging evidence, in particular, that this region and its connections have key roles in multiple functions, such as cognitive control and schema usage (Fernández, 2017; Metzler-Baddeley et al., 2012; Shenhav et al., 2013; Van Kesteren et al., 2013; Weible, 2013) that impact on memory. At the same time, the anterior cingulate region and its connections remain strongly implicated in emotional processes (Dalgleish, 2004; Etkin et al., 2011, 2015; Fan et al., 2011).

### Emotion and psychiatry

Despite the increased emphasis on memory in recent decades, the notion that the hippocampal–diencephalic–cingulate network (Papez, 1937) is vital for emotion never fully disappeared. From the 1950s to the 1980s, ideas about the limbic system remained centred on its likely role in emotion and its presumed imbalance in psychiatric conditions (Kelly, 1973; Livingston and Escobar, 1971). This preserved emphasis partly reflected how the term ‘limbic system’ had been broadened to include areas such as the amygdala and orbitofrontal cortex. Reflecting this trend, it was proposed that that the limbic system should be subdivided (Livingston and Escobar, 1971; Rolls, 2015). One subsystem was a ‘medial limbic’ circuit (Livingston and Escobar, 1971), which particularly emphasised anterior thalamic–posterior cingulate–hippocampal connections, that is, the connections highlighted in this review. This ‘medial limbic’ circuit was contrasted with an amygdala-based ‘basolateral circuit’ that included the anterior cingulate region, the two circuits jointly contributing to affect and learnt emotion (Dalgleish, 2004; Livingston and Escobar, 1971).

Within such conceptual frameworks, surgeons targeted sites such as the anterior cingulate cortex for obsessional (obsessive–compulsive disorder (OCD)) and affective disorders (Feldman et al., 2001; Lewin, 1961). Likewise, the cingulum bundle has been selectively damaged as a means to combat a variety of severe refractory psychiatric illnesses, including depression, anxiety, OCDs, and schizophrenia (Ballantine et al., 1987; Feldman et al., 2001). The literature fails to clarify or explain the specific resultant effects on cognition and behaviour and suggests simply that the disconnection of limbic structures from the forebrain disrupts the behavioural expression of internal emotional states (Ballantine et al., 1967). Nonetheless, it is noteworthy that memory deficits are not normally reported with such procedures. Another target, especially for OCD, has been the region of the anterior capsule at the anterior limb of the internal capsule (Feldman et al., 2001; Mashour et al., 2005). Such surgeries would be expected to disconnect thalamic–frontal pathways, including those anterior thalamic efferents that reach the cingulum in this way. At the same time, posterior cingulectomy (the removal of the posterior cingulate gyri) has been used for psychiatric conditions (Turner, 1973). The reports emphasise changes in emotion rather than memory (Turner, 1973). A feature of these various surgeries is that in different ways, they disrupt aspects of both the ‘medial’ (hippocampal–diencephalic–cingulate) and ‘basolateral’ limbic circuits.

In recent years, there has been renewed interest in the hippocampus and emotion, partly from growing evidence that hippocampal dysfunctions contribute to conditions such as schizophrenia, anxiety disorders, and posttraumatic stress disorder (PTSD; Small et al., 2011). This interest has been fuelled by the discovery of functional changes along the long axis of the hippocampus, which partly reflect changing relative contributions to emotion and memory (O’Mara et al., 2001; Poppenk et al., 2013; Small et al., 2011). In particular, it is supposed that the functions of the anterior hippocampus are biased towards emotional states, including anxiety, while the posterior hippocampus is more critical for mnemonic functions (Aggleton, 2012; McHugh et al., 2004; O’Mara et al., 2001; Ranganath and Ritchey, 2012; Small et al., 2011). This framework is reflected in hippocampal connectivity as the anterior hippocampus is particularly linked to sites such as the amygdala, nucleus accumbens, medial, and orbital prefrontal cortex, while the posterior hippocampus is more densely connected with sites closely linked to episodic memory, including the mammillary bodies and retrosplenial cortex (Aggleton, 2012). For this reason, the hippocampal–diencephalic–cingulate network appears to particularly engage the posterior hippocampus.

A related issue is that many of the hippocampal projections most associated with emotion do not, in fact, join those connections highlighted by Papez (Clarke et al., 2015; Small et al., 2011), as there are direct hippocampal projections to sites such as the frontal cortex, amygdala, and ventral striatum (Aggleton, 1986; Aggleton et al., 2015; Friedman et al., 2002). These particular hippocampal connections have been especially linked to conditions such as PTSD and schizophrenia (Sigurdsson and Duvarci, 2015; Small et al., 2011). Even so, animal studies reveal contributions from the anterior thalamic nuclei and retrosplenial cortex to fear conditioning (Célérier et al., 2000; Gabriel, 1991; Gabriel et al., 1991; Keene and Bucci, 2008), suggesting that Papez’s connections retain a contributory role in emotional conditions such as anxiety.

### Summary

In conclusion, we can state that the concept of a serial limbic circuit for emotion, first promoted by Papez (1937), is misleading with respect to both information flow and function. It remains the case that the connections originally described by Papez do exist. Indeed, it could be argued that with respect to the mammillary bodies, the anterior thalamic nuclei, and the retrosplenial cortex, these interconnections may well be the most dominant with respect to their respective functions. At the same time, Papez could not appreciate the weight of reciprocal connections between some of the structures, as well as the number of additional connections that jump the nodes in his circuit. This network, which appears more critical for learning and memory than emotion, involves complex topographies that reflect multiple subsystems. Furthermore, the predominant pattern of information flow need not be circular, as initially supposed. Instead, many of the connections can be seen as providing parallel efferents from the medial temporal lobes, where the subiculum has a particularly important role.

Despite all these complexities, the structures initially highlighted by Papez still retain a special status. One unifying example is that theta-rhythm appears to resonate throughout these same sites, consistent with a circuit (Vertes et al., 2001, 2004, 2015). Such neuronal activity potentially plays an important role in mnemonic processes. Another example is the way in which sites throughout the hippocampal–diencephalic–cingulate network contain head-direction cells (Taube, 2007). Furthermore, when trying to understand the relationships between conditions such as temporal lobe amnesia and diencephalic amnesia, or when trying to unravel the neuropathologies underlying prodromal states in dementia, the importance of these same structures and their interlinking pathways comes to the fore. Consequently, we still need a more comprehensive appreciation of the group of connections initially described 80 years ago by Papez, combined with the specific need to uncover far more about the details of these same connections in the human brain.
